# Self-Assembled Nanobodies as Selectively Targeted, Nanostructured, and Multivalent Materials

**DOI:** 10.1021/acsami.1c08092

**Published:** 2021-06-15

**Authors:** Laura Sánchez-García, Eric Voltà-Durán, Eloi Parladé, Elisa Mazzega, Alejandro Sánchez-Chardi, Naroa Serna, Hèctor López-Laguna, Mara Mitstorfer, Ugutz Unzueta, Esther Vázquez, Antonio Villaverde, Ario de Marco

**Affiliations:** † Institut de Biotecnologia i de Biomedicina, Universitat Autònoma de Barcelona, Bellaterra, Barcelona 08193, Spain; ‡ Departament de Genètica i de Microbiologia, Universitat Autònoma de Barcelona, Bellaterra, Barcelona 08193, Spain; § CIBER de Bioingeniería Biomateriales y Nanomedicina (CIBER-BBN), Bellaterra, Barcelona 08193, Spain; ∥ Laboratory for Environmental and Life Sciences, University of Nova Gorica Nova Gorica 5000, Slovenia; ⊥ Servei de Microscòpia, Universitat Autònoma de Barcelona, Bellaterra, Barcelona 08193, Spain; # Departament de Biologia Evolutiva, Ecologia i Ciències Ambientals, Facultat de Biologia, Universitat de Barcelona, 08028 Barcelona, Spain; ∇ University of Natural Resources and Life Sciences, Department of Chemistry, Institute of Biochemistry, 1190 Vienna, Austria; ¶ Biomedical Research Institute Sant Pau (IIB Sant Pau), Sant Antoni Ma̲ Claret 167, 08025 Barcelona, Spain

**Keywords:** nanobodies, self-assembling, ricin, nanoparticles, controlled delivery, biomaterials

## Abstract

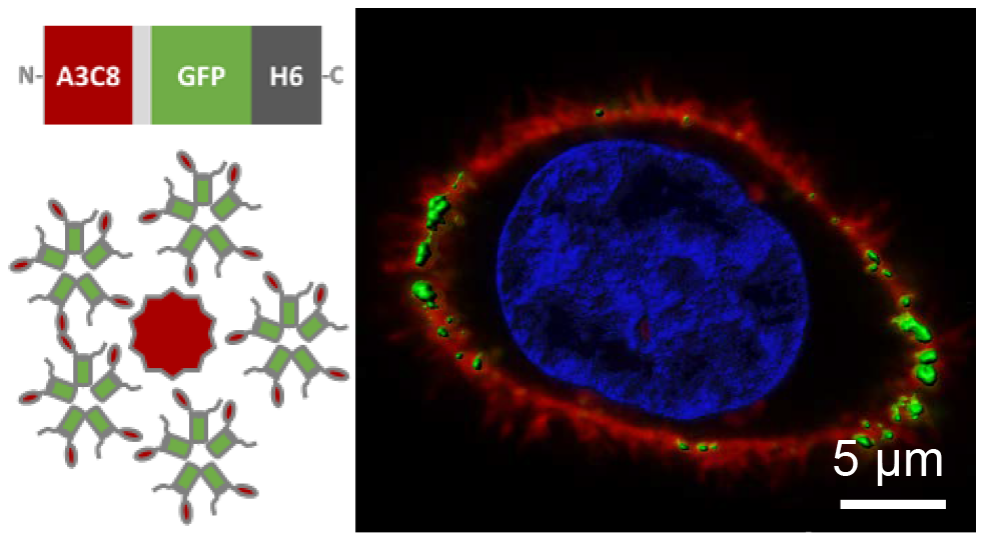

Nanobodies represent valuable tools in advanced therapeutic strategies but their small size (∼2.5 × ∼ 4 nm) and limited valence for interactions might pose restrictions for *in vivo* applications, especially regarding their modest capacity for multivalent and cooperative interaction. In this work, modular protein constructs have been designed, in which nanobodies are fused to protein domains to provide further functionalities and to favor oligomerization into stable self-assembled nanoparticles. The nanobody specificity for their targets is maintained in such supramolecular complexes. Also, their diameter around 70 nm and multivalent interactivity should favor binding and penetrability into target cells via solvent-exposed receptor. These concepts have been supported by unrelated nanobodies directed against the ricin toxin (A3C8) and the Her2 receptor (EM1), respectively, that were modified with the addition of a reporter protein and a hexa-histidine tag at the C-terminus that promotes self-assembling. The A3C8-based nanoparticles neutralize the ricin toxin efficiently, whereas the EM1-based nanoparticles enable to selective imaging Her2-positive cells. These findings support the excellent extracellular and intracellular functionality of nanobodies organized in form of oligomeric nanoscale assemblies.

## Introduction

The selective delivery of molecules to target cells or organs is critical to improve the specificity of therapy and image-based diagnosis. Molecular ligands are able to specifically recognize and bind cell-surface biomarkers overexpressed in target cells and they can be then used to confer selectivity to drugs, imaging agents, or combined with supramolecular complexes. Proteins, peptides, antibodies, polysaccharides, aptamers, and a variety of small molecules have been explored and developed as targeting ligands and their comparative potentialities and drawbacks have been deeply described.^1^

Antibodies have evolved to recognize their antigens with high affinity and specificity, and therefore, they represent the most common class of binders used in both research and clinical settings. Specifically, conventional IgG monoclonal antibodies issued from hybridoma cells are currently the most preferred antibody format. However, their large mass (150 kDa) and structural complexity make them not suitable for some applications such as the targeting of solid tumors. These properties also hamper the access to dense structures such as tumoral tissues while increasing the probabilities to interact with other molecules in the bloodstream, to accumulate in liver and to induce immunogenicity. In addition, their clonality is often unstable, their engineering is complex, their functionalization not reproducible and they are expensive and difficult to produce due to their highly glycosylated domains.^2^ The use of IgG fragments (Fab and scFv formats) can fundamentally solve most of these problems but introduces others, such as a reduced avidity when compared to multivalent alternatives and higher propensity to aggregate.^3^

**Figure 1:**
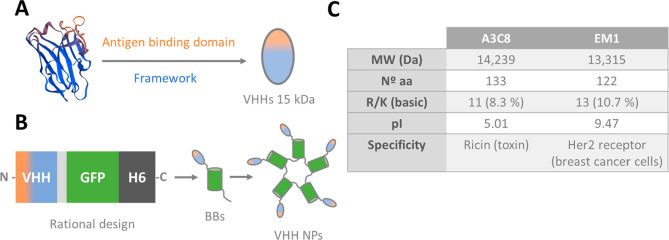
Physicochemical properties and structure of nanobodies. A. Representation of the generic nanobody structure. The conserved region (framework) is shown in blue and the variable antigen-binding domain (comprising CDR 1, 2, and 3) in orange. B. Design and formation of VHH self-assembled nanoparticles. C. Properties of selected nanobodies A3C8 and EM1. Abbreviations: BBs: building-blocks, MW: molecular weight, aa: amino acids, R: arginine, K: lysine, and pI: isoelectric point.

In this scenario, the discovery of camelid heavy-chain antibodies (HcAbs) opened interesting possibilities.^4^ Unlike conventional antibodies or IgG fragments, the variable antigen-binding fragment of HcAbs consists of a single structural domain (15 kDa), known as nanobody or VHH (variable heavy chain of HcAbs) (Figure 1A). It corresponds to an amino acid sequence that can be easily produced by recombinant DNA procedures and that preserves the binding selectivity of the whole molecule. The properties of nanobodies, such as small size, good stability and ease of engineering and expression made them the preferred components in several biotechnological applications.^5−7^ They have been successfully produced alone or as fusion proteins in Gram-negative (*Escherichia coli)* and Gram-positive bacteria (*Lactobacillus* sp), yeasts, plants, and mammalian cells, according to the required structural and functional features of the final product.^8,9,7^ Additionally, VHHs are known to be poorly immunogenic and their immunogenicity can be further reduced by site-directed mutagenesis.^5^

In February 2019, the Food and Drug Administration (FDA) approved the use of the first nanobody (Caplacizumab). Caplacizumab is a recombinant humanized nanobody produced in *E. coli*(10) and used as a drug for the treatment of acquired thrombotic thrombocytopenic purpura (aTTP), a rare disease characterized by excessive blood clotting in small blood vessels.^11^ Other nanobodies have reached different phases of clinical trials, such as Ozoralizumab NCT01007175 and Vobarilizumab NCT02287922 (to treat rheumatoid arthritis), M1095 NCT02156466 (for psoriasis) and BI 836880 NCT03468426 (for solid tumors), evidencing the potential of nanobodies in different therapeutic areas.^12^

As stated above, the low molecular mass of nanobodies facilitates their tissue penetration.^13^ However, in contrast, the cellular interactivity of VHHs is highly limited by their small size (around 4 nm),^14,15^ far from optimal values around 20–80 nm.^16,17^ In this context, different strategies have been developed aiming at enlarging nanobodies by their combination with other molecules.^7,18−23,23^ Larger structures (around 24 nm) have been indeed obtained by fusing VHHs to elastin-like peptides and by linking VHHs to the pentameric B-subunit of *E. coli* verotoxin.^24−26^ Also, the genetic fusion of the albumin-binding domain allows a later incorporation of albumin as an external agent for size increase.^27^ Ideally, size increase should be achieved by oligomerization rather that by the coupling VHHs to any heterologous material. In this way, the chemical homogeneity of the material would be conserved and reproducible, and its functionality would be exploited in full. Under this concept, the engineering of VHHs by gene fusion to generate modular proteins would confer them the capability to self-assemble in form of nanoparticles, if proper protein domains, favoring self-assembling, are selected for the construct. This approach would not only contribute to increase the mass of a VHH but also promote a multivalent presentation of the ligand. Such a modular design based on domain recruitment might also permit selecting fusion partners with particular functions that would be combined in single multifunctional particulate entities keeping the precise interactivity of the nanobody.

In this context, we have recently generated several categories of protein-only self-assembling nanoparticles following a semirational protein engineering strategy.^28−30^ According to this approach, the combination of N-terminal domains with cationic character with C-terminal histidine-rich peptides (such as the hexahistidine H6) favors spontaneous oligomerization into regular nanoparticles through a particular distribution of electrostatic charges.^29,31^ Apart from other protein–protein interactions that contribute to the assembly, such as hydrogen bond and van der Waals,^28^ divalent cations present in the media stabilize such complexes^32^ through their coordination with overhanging H6 tails.^33−35^ No external addition is needed for such stabilization,^36^ as Ni^2+^ traces from the purification columns might be sufficient.^33,34^ Because of the β barrel folding of GFP, GFP-containing constructs are particularly suited for fast and efficient nanoparticle formation following this principle, as proved by diverse categories of fusion proteins of biomedical interest that are based on H6-tagged GFP.^37−39^ This engineering approach based on self-oligomerization allows enlarging the size of the materials by reaching a multimeric organization and it prevents the introduction of irrelevant protein domains or other scaffold materials that might represent a load in production and a risk regarding the toxicity of the final hybrid material.^40^ Therefore, we decided to explore the short H6 tag, fused to nanobodies and GFP (Figure 1B), as a tool to generate large self-assembled multivalent and multifunctional nanoparticles. For that, two unrelated nanobodies were selected as models, namely A3C8, specific for ricin toxin, and EM1, specific for Her2 (Figure 1C),^41,42^ and engineered to demonstrate the feasibility of the proposed approach, that is, controlled oligomerization into nanoparticles while preserving functionalities of the nanobodies and of other incorporated protein domains.

## Materials and Methods

### Protein Design, Production, and Purification

**Figure 2:**
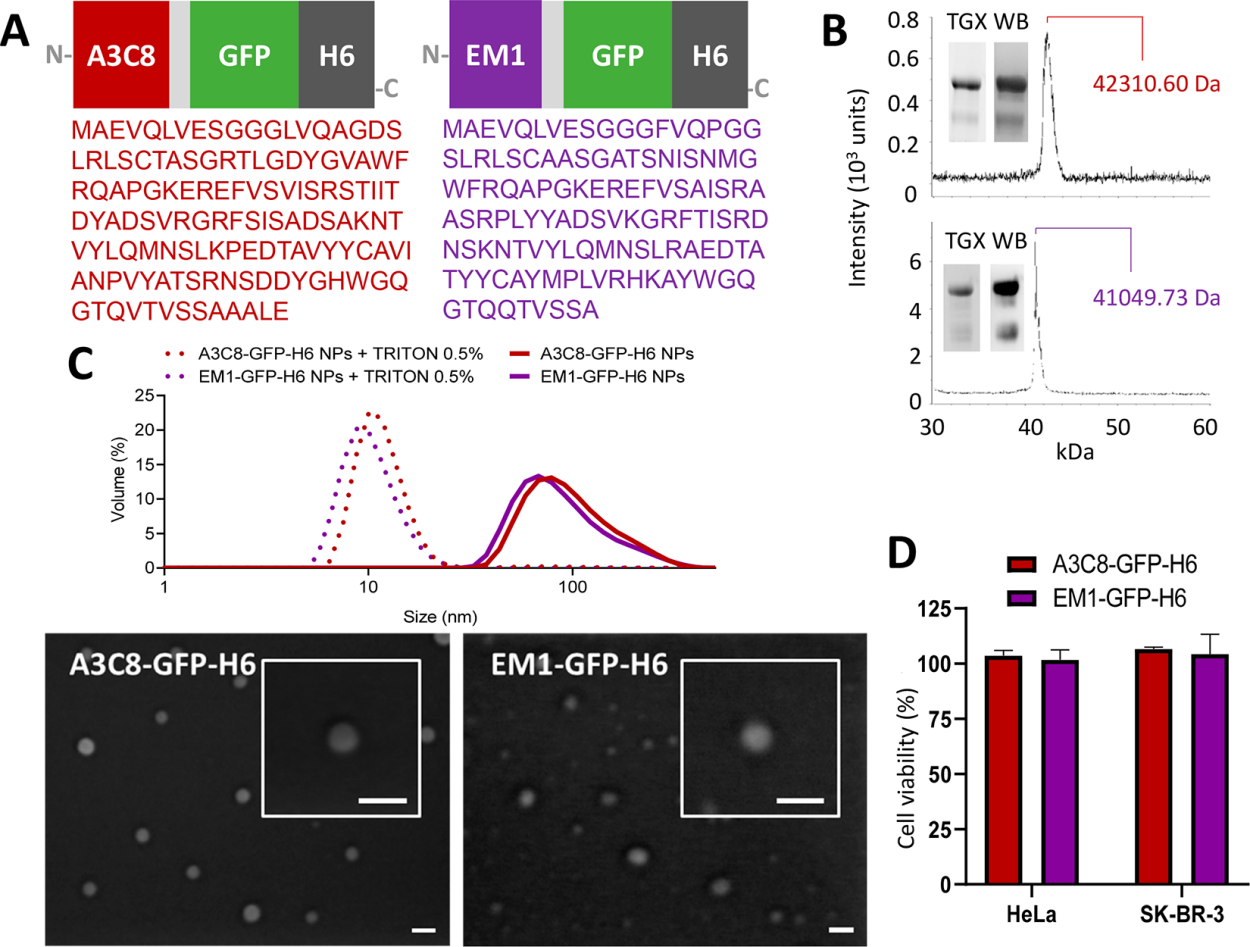
Physicochemical characterization of VHH-based modular proteins. A. Modular organization of VHHs fused to GFP-H6 and their corresponding amino acid sequence. VHHs A3C8 and EM1 (in dark red and purple, respectively) serve as selective ligands. A short linker (GGSSRSS) in light gray was added between the VHH and GFP for conformational purposes. GFP protein (in green) was incorporated for tracking purposes while H6 (in dark gray) for purification and for assembling purposes. Box sizes are only indicative. B. MALDI-TOF, SDS-PAGE (TGX), and Western Blot (WB) of purified recombinant proteins. Molecular weights are indicated. C. Dynamic light scattering (top) and FESEM images (bottom) of purified assembled materials. Triton was used for disassembling at 0.5%. Bars size: 100 nm. D. Cell viability of HeLa and SK-BR-3 cells after 72 h protein incubation at 1 × 10^–7^ M.

The modular constructs A3C8-GFP-H6 and EM1-GFP-H6 (Figure 2A) were designed in-house as *E. coli* codon-optimized genes and synthesized by GeneArt (ThermoFisher). The nomenclature of fusion proteins used in this study refers to their modular domain organization. A3C8 is a nanobody with high affinity toward the plant toxin ricin, whereas EM1 is specific for Her2 receptor, overexpressed in breast cancer cells. Green fluorescent protein (GFP) was incorporated in the constructs for tracking purposes, and the C-terminal hexa-histidine tag (HHHHHH) was added for cation-coordinated protein oligomerization and for purification purposes.^33^ A flexible peptide (GGSSRSS) connecting nanobody and GFP protein was introduced as a spacer to impair undesired structural interactions between the moieties that could interfere with their functionality. Both fusion sequences A3C8-GFP-H6 and EM1-GFP-H6 were subcloned in the plasmid pET22b and further transformed by heat shock (42 °C for 45 s) in *Escherichia coli* Origami B (BL21, OmpT–, Lon–, TrxB–, Gor−) (Novagen, Germany).

Transformed cells (selected through the acquisition of ampicillin resistance provided by pET22b) were incubated at 37 °C until reaching OD_550_ = 0.5–0.7. Then, protein production was performed at 20 °C overnight after the addition of IPTG (isopropyl β-d-1-thiogalactopyranoside) at a concentration of 0.1 × 10^–3^ M (A3C8-GFP-H6) and 1 × 10^–3^ M (EM1-GFP-H6). Finally, cells were centrifuged (5000*g* for 15 min 4 °C) and stored at −80 °C until use. Pellets were thawed and resuspended in Wash buffer (20 × 10^–3^ M Tris-HCl, 500 × 10^–3^ M NaCl, 10 × 10^–3^ M imidazole, pH 8.0) supplemented with the protease inhibitor cOmplete EDTA-free (Roche, U.S.). Bacterial disruption was performed in a French Press at 1200 psi (Thermo FA-078A) for optimal lysis. The soluble fraction was collected after centrifugation (45 min, 15 000*g* at 4 °C) and filtered through a 0.22 μm-diameter filter. Protein purification was performed in an Äkta Pure FPLC system (GE Healthcare, U.S.) by Immobilized Metal Affinity Chromatography (IMAC). After the selective binding of the protein onto a HisTrap HP column (GE Healthcare, U.S.), elution was achieved by applying a linear gradient of Elution buffer (20 × 10^–3^ M Tris-HCl, 500 × 10^–3^ M NaCl, 500 × 10^–3^ M imidazole, pH 8.0). The eluted samples were dialyzed against sodium carbonate salt buffer (166 × 10^–3^ M NaCO_3_H and 333 × 10^–3^ M NaCl, pH 8.0) and centrifuged to remove protein aggregates (15 min, 15 000*g* at 4 °C). Protein quantification was performed using Bradford Assay (BioRad, U.S.).

### Physicochemical Characterization

A3C8-GFP-H6 and EM1-GFP-H6 proteins were analyzed to assess their degree of purity by SDS-PAGE using TGX Stain-Free FastCast gels (BioRad, U.S.). Protein integrity was also assessed by Western-blot using an anti-His monoclonal antibody (Santa Cruz Biotechnology, U.S.), and by mass spectrometry (MALDI-TOF). Additionally, the volume size distribution was determined by dynamic light scattering (DLS) at 633 nm (Zetasizer Nano ZS, Malvern Instruments Limited, UK), in 50 μL of protein storage buffer (in of 166 × 10^–3^ M NaCO_3_H and 333 × 10^–3^ M NaCl). For disassembling, proteins were diluted at 0.5 mg mL^–1^, and Triton X-100 added to 0.5% was used in order to visualize their respective building blocks by DLS. Size measurements were performed in triplicate. Volume data in DLS was representative of the population size distribution while Intensity data provided more accurate hydrodynamic sizes.

### Ultrastructural Characterization

Ultrastructural morphometry (size and shape) of nanoparticles was visualized at nearly native state with field emission scanning electron microscopy (FESEM) and transmission electron microscopy (TEM). Drops of 5 μL of nanoparticles resuspended in their buffers were directly deposited on silicon wafers (Ted Pella Inc.) for 2 min, excess of liquid blotted, air-dried, and immediately observed without coating in a FESEM Merlin (Zeiss) operating at 1 kV and equipped with an in-lens secondary electron detector. Drops of 5 μL of nanoparticles resuspended in their buffers were also deposited on carbon-coated copper grids (400 mesh, Agar Scientific) for 2 min, excess of liquid blotted, incubated in 2% uranyl acetate (Sigma-Aldrich, Germany) for 5 min, rinsed with deionized water, and air-dried. Samples were observed in a TEM JEM-1400 (Jeol Ltd.) operating at 80 kV and equipped with an Orius SC 200 CCD camera (Gatan). Representative images of general fields and nanostructure details were captured at two high magnifications (2 × 10^5^× and 5× 10^5^× for FESEM, and 1× 10^4^x and 8× 10^4^× for TEM).

### Cell Culture and Flow Cytometry

Human cervical adenocarcinoma cells (HeLa cell line, ATCC, CCL-2) were maintained in MEM-Alpha (Gibco) and incubated at 37 °C and 5% CO_2_ in a humidified atmosphere. Human breast cancer cells (SK-BR-3 cell line, ATCC, HTB-30) were maintained in DMEM (Gibco) at 37 °C and 10% CO_2_. Both culture media were supplemented with 10% fetal bovine serum (Gibco). HeLa cells (Her2^–^) and SK-BR-3 cells (Her2^+^) were scattered in 24-well plates at 6 × 10^4^ and 8 × 10^4^ cells/well, respectively. After 24 h, cells reached 70% confluence and were incubated with 1000 nM of EM1-GFP-H6 for 24 h. Two different trypsinization protocols (harsh and mild) were compared to detach the cells and analyze them through flow cytometry.^43^ A harsh trypsin (HT) protocol (1 mg mL^–1^ for 15 min) was aimed to remove cells from the well while stripping the residual protein attached on the cell membrane surface. Mild trypsin (MT) protocol (0.5 mg mL^–1^ for 5 min) was conceived to detach cells from the well without removing bound protein from the cell surface. Experiments were performed in duplicate.

### Cell Viability and Neutralization Assay

The CellTiter-Glo Luminescent Cell Viability Assay (Promega) was used to determine the cytotoxicity of the purified recombinant proteins and the neutralizing effect of A3C8-GFP-H6 nanoparticles. HeLa cells (CXCR4^+^) and SK-BR-3 cells (Her2^+^) were scattered at 3500 cells/well in opaque-walled 96-well plates, until reaching 70% confluence. To assess whether A3C8-GFP-H6 and EM1-GFP-H6 nanoparticles are cytotoxic, HeLa cells and SK-BR-3 cells were treated with a final concentration of 1 × 10^–7^ M for 72 h. In the neutralization assay, ricin toxin (1 × 10^–8^ M of the recombinant T22-mRTA-H6) was preincubated with increasing amounts of A3C8-GFP-H6 at different ratios (1:1, 1:5, and 1:10) for 72 h. The irrelevant construct EM1-GFP-H6 (anti-Her2 nanoparticle) was used as a negative control.^28^ After protein incubation, the reagent provided by the manufacturer was added to cultured cells and plates were measured in a conventional luminometer Victor3 (PerkinElmer, U.S.). Experiments were performed in triplicate.

### Confocal Laser Scanning Microscopy

HeLa and SK-BR-3 cells were grown on Mat-Tek plates (MatTek Corporation, U.S.) scattering 1.2 × 10^5^ cells per well. After cellular attachment, protein incubation was performed in the presence of EM1-GFP-H6 or GFP-H6 at a final concentration of 1 μM for 1 h. After protein incubation, cell nuclei were labeled with 5 μg mL^–1^ Hoechst 33342 (ThermoFisher, U.S.) and the plasma membrane with 2.5 μg mL^–1^ CellMask Deep Red (ThermoFisher, U.S.) for 10 min at room temperature. Cells were then washed in DPBS buffer (Sigma-Aldrich, Germany). All confocal images were collected on an inverted TCS SP5 Leica Spectral confocal microscope (Leica Microsystems, Germany) using 63× (1.4 NA) oil immersion objective lenses. Excitation was reached using a 405 nm blue diode laser (nucleic acids), 488 nm line of an argon ion laser (nanoparticles) and 633 nm line of a HeNe laser (cell membrane). Optimized emission detection bandwidths were configured to avoid interchannel crosstalk and multitrack sequential acquisition setting were used. The confocal pinhole was set to 1 Airy unit and z-stacks acquisition intervals were selected to satisfy Nyquist sampling criteria. Three-dimensional images were processed using the Surpass Module in Imaris X64 v.7.2.1. software (Bitplane, Switzerland).

### Statistical Analysis

The data of the in vitro experiments (cell viability and protein internalization) were reported as mean ± SEM (Standard Error of the Mean). Results were analyzed using Tukey’s pairwise test. Differences between groups were considered significant at *p* < 0.05. These differences were indicated as * *p* < 0.05 and ** *p* < 0.01. Statistical calculations were performed using Past3 software.

## Results

Recombinant fusion proteins formed by GFP-H6 and VHHs (A3C8 and EM1) as targeting moieties (Figure 2A) were successfully produced in *E. coli*. After purification, protein purity was assessed by SDS-PAGE and protein integrity was confirmed by MALDI-TOF and Western Blot (Figure 2B). The molecular weights of A3C8-GFP-H6 and EM1-GFP-H6 were 42.31 and 41.05 kDa, respectively, in agreement with the theoretically calculated values.

Both VHHs are rich in arginine and lysine residues (over 5%, Figure 1C,A), and we wondered if the positive charge conferred by these residues would be enough to enable the protein to self-assemble, as demonstrated for other H6-tagged fusion proteins.^29^ While a fraction of the protein was purified from bacterial cell extracts as monomers (with an hydrodynamic size around 8 nm, not shown), dynamic light scattering (DLS) revealed that most of A3C8-GFP-H6 and EM1-GFP-H6 in the eluted protein fraction self-assembled, as expected, into nanoparticles of around 70 nm (Figure 2C). Upon detergent-induced nanoparticle disassembly, protein materials of around 10 nm in diameter were detected (Figure 2C), compatible with protein forms that represented the building blocks in the protein oligomerization process. Protein nanoparticles showed a regular pseudospherical shape, as observed by FESEM (Figure 2C), and their dimensions, determined by FESEM, were in full agreement with DLS volume determinations. Then, self-assembled nanobodies were studied *in vitro* for their physiological properties. Cell viability experiments in HeLa (Her2^–^) and SK-BR-3 (Her2^+^) mammalian cells showed, as expected, no cytotoxic effect after 72 h of incubation with any of the nanoparticles (Figure 2D), indicating that the oligomerization event did not confer toxicity to proteins that are intrinsically innocuous.

At this point, we assessed the capacity of nanoparticles to bind to their target antigens. First, the capacity of A3C8-GFP-H6 to recognize and neutralize ricin was demonstrated. Ricin toxin consists of two chains linked by a disulfide bond. The chain A (RTA) corresponds to the catalytic domain with N-glycosidase enzymatic activity, whereas the chain B (RTB) corresponds to the carbohydrate recognition protein. It has been described that RTB cell-binding is unspecific and presents low affinity, promoting multiple intracellular trafficking pathways.^44^ In order to obtain a finely controlled and highly specific version of the toxin, we selected a previously designed and fully active recombinant chimera (T22-mRTA-H6) composed of a mutant A chain (mRTA) fused to the N-terminal CXCR4-binding peptide T22^45^ and to a C-terminal histidine-rich tag.^46^ The CXCR4-binding peptide confers specificity for such receptor whereas the poly-His, as described above, contributes to the oligomerization of the construct. Therefore, the recombinant T22-mRTA-H6, that self-assembled as potent cytotoxic nanoparticles of 11 nm, selectively kills CXCR4^+^ cells in vitro and in vivo^45^ and is then usable to assess the protective effect of the antiricin A3C8-GFP-H6 nanoparticles over cultured CXCR4^+^ HeLa cells.

**Figure 3:**
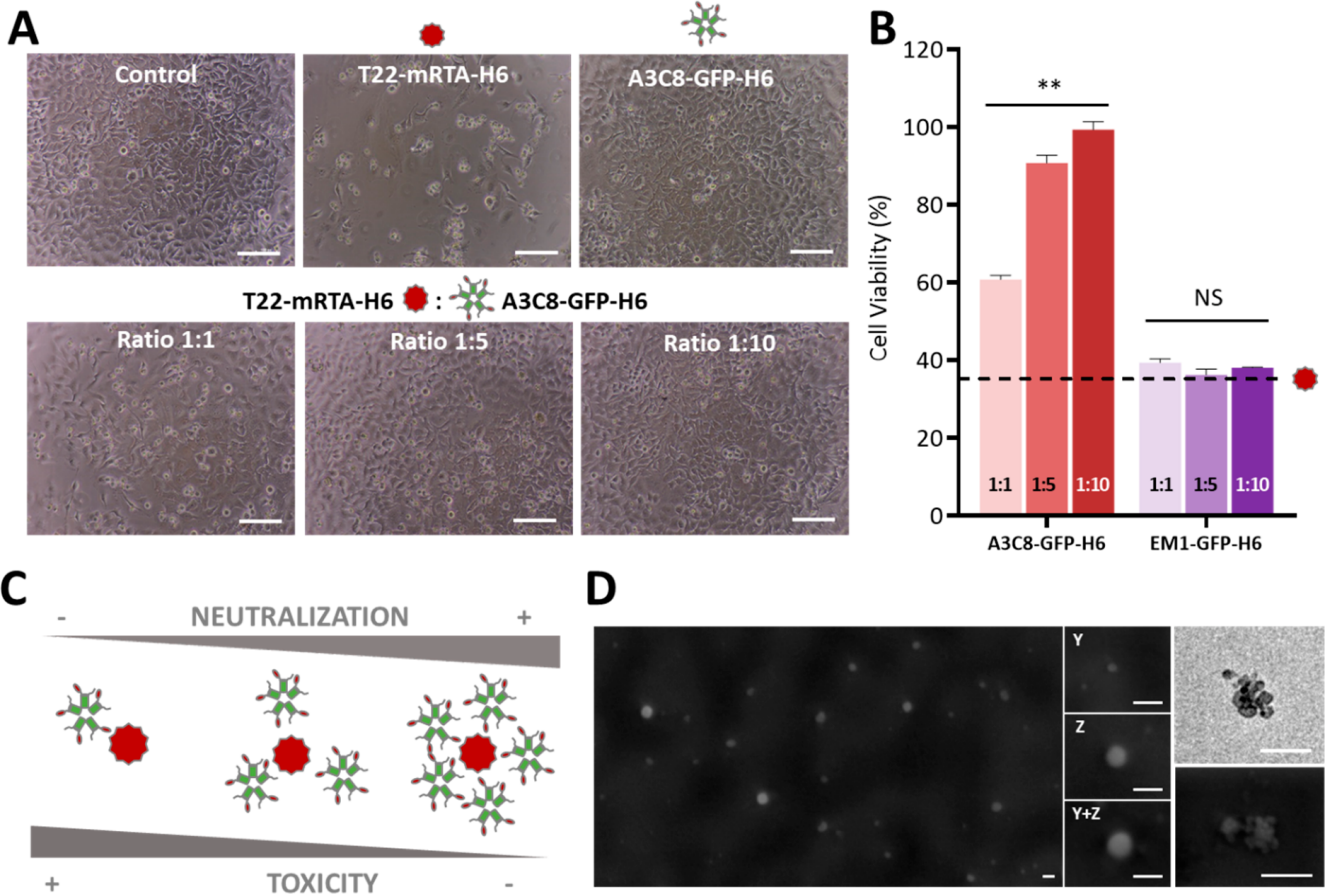
Neutralization capacity of antiricin A3C8-GFP-H6 nanoparticles. A. Optical microscopy images of HeLa cells treated with 1 × 10^–8^ M T22-mRTA-H6 and 1 × 10^–7^ M A3C8-GFP-H6 separately (top) and in combination at ratio 1:1, 1:5, 1:10 (bottom). Bars size: 100 μm. B. Quantitative data of the neutralization assay performed at 72 h expressed as percentage of cell viability. EM1-GFP-H6 is used as a negative control. Dashed line illustrates cell viability after treatment with 1 × 10^–8^ M T22-mRTA-H6 under the same conditions. Significant differences between each particular condition and T22-mRTA-H6 alone are indicated as ** *p* < 0.01. NS: not significant. C. Schematic representation of the neutralization assay and the phenomenon occurring at the different conditions. D. Representative electron microscopy (FESEM and TEM) images of T22-mRTA-H6 toxin treated with A3C8-GFP-H6 showing nanoparticle populations classified by size as free T22-mRTA-H6 (Y), A3C8-GFP-H6 (Z), and complex of the two components (Y+Z). Aggregates compatible with few A3C3-GFP-H6 neutralizing free ricin were shown at right. Bars size: 100 nm.

The neutralizing activity of A3C8-GFP-H6 was demonstrated by measuring the CXCR4^+^ HeLa cell viability at increasing concentrations of the antidote with reference to those of the toxin (T22-mRTA-H6:A3C8-GFP-H6 molar ratio of 1:1, 1:5, 1:10) (Figure 3). Optical microscopy images showed that cell confluence was proportional to the increase of neutralizing nanoparticles (Figure 3A). Precisely, cell viability increased from 35% up to 60% when neutralizing nanoparticles were used at 1:1 ratio with respect to the toxin alone (T22-mRTA-H6) and reached 100% at a 1:10 ratio (Figure 3B). Self-assembled control EM1-GFP-H6 nanoparticles were instead unable to neutralize the ricin-induced cytotoxicity at any concentration (Figure 3B). Moreover, FESEM and TEM imaging revealed that the coincubation of nanobody and ricin nanoparticles originated a new population of larger nanoparticles (Figure 3C,D), with a size compatible with agglutinated nanobody and ricin constructs.

**Figure 4:**
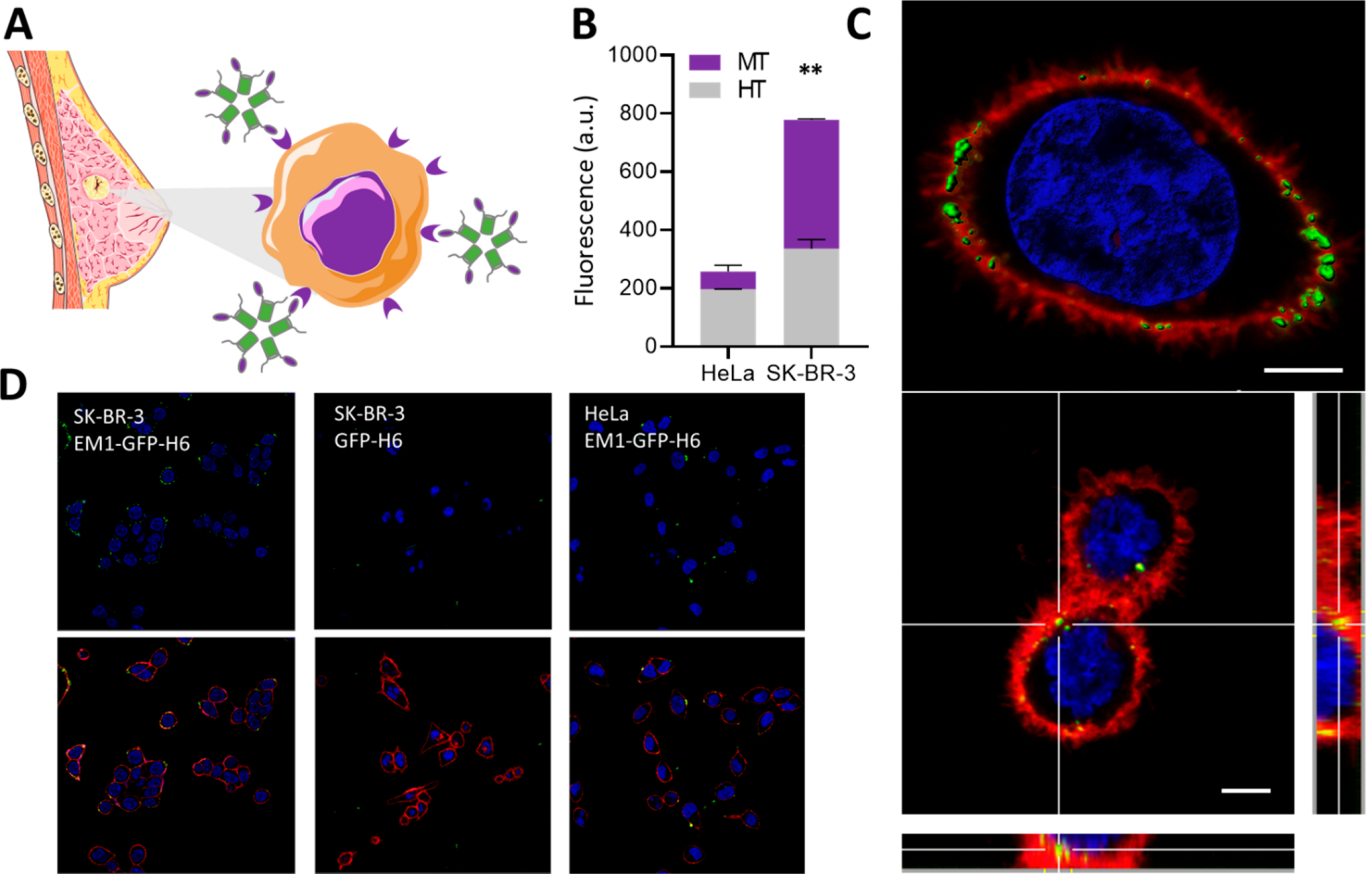
In vitro assessment of EM1-GFP-H6 binding to Her2 receptor. A. Schematic representation of Her2-targeted nanoparticles (EM1-GFP-H6) binding to Her2^+^ breast cancer cells (sizes are not representative). B. Flow cytometry of EM1-GFP-H6 nanoparticles in Her2^+^ (SK-BR-3) and Her2^–^ (HeLa) cells. HT and MT correspond to harsh and mild trypsin protocols, respectively. Significant differences are indicated as ** *p* < 0.01. C. Confocal images of SK-BR-3 (Her2^+^) cells after incubation with 1 μM of EM1-GFP-H6 for 1 h. Red signal corresponds to cell membrane, blue to nuclei and green to nanoparticles. At the bottom, orthogonal projections show the localization of EM1-GFP-H6 nanoparticles in yellow, due to the colocalization of red (membrane) and green (nanoparticle) signal. Bars size: 5 μm. D. Wide confocal fields of cultured SK-BR-3 cells and HeLa cells exposed to EM1-GFP-H6 nanoparticles and to a control GFP-H6 protein. Cells are shown without (top) and with (bottom) membrane staining.

The capacity of EM1-GFP-H6 to bind to the Her2 receptor (Figure 4A) was tested by flow-cytometry using Her2^–^ (HeLa) and Her2^+^ cells (SK-BR-3) (Figure 4B). Both cell lines were first incubated with the nanoparticles followed by trypsinization. Two alternative treatments, referred to as mild trypsin (MT) and harsh trypsin (HT), were applied to distinguish between fluorescence located at the external cell surface or due to nanoparticle internalization. Her2^–^ HeLa cells exposed to nanoparticles only acquired a low background signal, which indicated negligible nanoparticle binding to both cell subgroups (MT and HT). In contrast, the incubation of nanoparticles with Her2^+^ cells resulted in a significant increase of the fluorescence linked to these cells. The strong effect of harsh trypsinization (Figure 4B) suggested that EM1-GFP-H6 nanoparticles were mostly located at the level of the cellular membrane. Their precise localization was assessed by confocal microscopy (Figure 4C), and the specificity of the cellular interaction fully confirmed by the absence of green signals in Her2^–^ HeLa cells exposed to EM1-GFP-H6 and in Her2^+^ cells exposed to a control GFP-H6 (Figure 4D). The images revealed that important amounts of EM1-GFP-H6 nanoparticles accumulated in the cell membrane, with preference for specific foci. However, a moderate green fluorescent signal was still observed in the cytoplasm indicative of internalization. The overall set of such data indicated that EM1-GFP-H6 nanoparticles efficiently and specifically interacted with the Her2 receptor, although its moderate cationic character, sufficient for assembling, might be an obstacle for efficient internalization.^47^

## Discussion

Llama-derived nanobodies are small-sized ligands easy to produce and engineer. Whereas their reduced dimension is very desirable for some applications, for others it would be preferable having enlarged versions and desirable, with multivalent interactivity. The results presented in this study illustrate the development of a new category of nanobody-based, self-assembled, multivalent nanoparticles with dimensions larger than the original nanobodies but with preserved antigen selectivity and newly acquired fluorescence. To demonstrate this concept, we used model nanobodies with specificity toward two targets of interest in nanomedicine and designed modular proteins that have been successfully produced, purified and functionally evaluated. The constructs consisted of a nanobody domain fused to GFP and a H6-tag. The fluorescent protein simplifies the construct visualization whereas the poly-His tag is exploited as a convenient tag for both protein purification and to assist assembling of the nanobody-based fusion protein. While some H6-tagged proteins require the incorporation of additional cationic N-terminal peptides to induce assembling,^29,48^ the occurrence of a few clustered cationic amino acids in the N-terminal domain of the selected VHHs (Figure 2A) was proved to be sufficient for oligomerization (Figure 2C). A similar event was demonstrated elsewhere when producing recombinant versions of H6-tagged microbial proteins.^36^ In the whole category of H6-tagged constructs, divalent cations, even in traces such as Ni^2+^ leaking from the purification columns, are proved to stabilize the oligomers through cross-interactions between adjacent solvent-exposed histidine-rich tails.^33^ Even for self-assembling constructs such as those based on N-terminal arginine-rich peptides (R6, R9, etc.),^29,48^ it has been shown that divalent cations have a stabilizing effect.^32,33^ Further details regarding the formation of stable divalent cation-mediated materials and the role of histidine-rich peptides in such cross-molecular interactions are available elsewhere.^34,35^

Most of the engineered VHHs molecules spontaneously self-assembled as 70 nm-sized nanoparticles upon purification, well above the renal filtration cutoff (around 8 nm), making them optimal for potential in vivo applications.^49^ In this form, the VHHs offer a multivalent presentation of the ligands that results in highly specific targeting, as demonstrated by Her2^+^ cell labeling by the GFP-containing anti-Her2 nanoparticles. The successful characterization of such nanomaterials in terms of structural and functional features opens the opportunity to test them in applications such as imaging or drug delivery, in which monomeric or multimeric VHHs have already provided promising results.^50−55^ While the targeting of EM1-GFP-H6 materials for the Her2 receptor is efficient and selective (Figure 4B–D), the internalization of this construct is only moderate (Figure 4C,D). Although specific cell labeling for diagnosis purposes is a main utility of nanobodies,^56^ these molecules have been also described as targeting agents in drug complexes intended for cell-targeted drug delivery.^56^ However, cell penetrability of nanobodies is a nonconsistent event regarding efficacy. It is dramatically enhanced by increasing the cationic character of these molecules through selective mutagenesis of solvent-exposed residues^47^ or by photochemical induction,^57^ among other strategies. While the nanobody domain of EM1-GFP-H6 has a significant positive charge (Figure 1C) sufficient for self-assembly, it might be insufficient for efficient internalization. Further protein engineering addressed to introduce additional cationic residues or tails should enhance cell penetrability.

On the other hand, a specific application for nanobody nanoparticles is the exploitation of their agglutinating capacity. Toxins are a current threat with a great daily impact, and a lot of effort is being invested toward the detection and treatment of intoxications caused by toxins (botulinum toxin, anthrax toxin, ricin, toxic-shock syndrome toxin-1, *Salmonella typhymurium* toxin) and venoms.^21,58−60^ Ricin, produced in castor beans, is one of the most lethal toxins in nature.^61^ The high toxicity and the large availability of castor beans has led to a significant number of human intoxications. Moreover, no postexposure therapeutics are available to reverse the effects of intoxication, which can lead to death from poisoning within 36–72 h of exposure. In this context, we have selected the A3C8 nanobody for the development of neutralizing antiricin self-assembled nanoparticles, which showed high potency against a ricin toxin chimera (T22-mRTA-H6). Even at the lowest tested molar ratio (1:1 ratio T22-mRTA-H6:A3C8-GFP-H6), a neutralization of 39% was observed, followed by an 86% at 1:5 ratio and a 99% of neutralization at 1:10 ratio. The use of a functionally unrelated control (EM1-GFP-H6) demonstrated that the neutralization was not mediated by the structure of the nanobody as a nanoparticle, but it is specifically due to the selective binding capacity of the A3C8 nanobody. The generation of antiricin A3C8-GFP-H6 nanoparticles opens a wide spectrum of applications in nanomedicine, as they may be used as diagnostic agents or be applied prior contact with the toxin to provide passive immunity. Finally, these materials could be administered as a neutralizing antidote to intoxicated patients when the toxin is already circulating in the bloodstream. Similarly, the same nanoparticle format could be used to agglutinate other targets by a similar engineering of the corresponding nanobodies.

## Conclusions

The rational design of two unrelated nanobodies (A3C8 and EM1) fused to a H6-tagged GFP has allowed us to obtain multivalent protein-only nanoparticles that preserve their intrinsic binding specificity. A simple recombinant DNA technology approach is then a suitable tool to obtain highly specific biocompatible oligomers in a single-step process. In this regard, other examples of self-assembling nanobodies have been previously developed following different strategies such as fusion to elastin-like peptides or naturally oligomeric proteins (B-subunit homopentamer from verotoxin).^24−26^ Nonetheless, the methodology proposed in the current study does not rely on the presence of any bulky domain that might pose structural limitations or safety or immunogenic issues (due to the presence of nonfunctional material) when moving to *in vivo* applications but in the self-assembling properties of specifically engineered nanobody versions.

## Abbreviations

DLS: dynamic light scattering
Fab: antigen-binding fragment
FDA: food and drug administration
FESEM: field emission scanning electron microscopy
GFP: green fluorescent protein
H6: hexahistidine peptide
HcAbs: camelid heavy-chain antibodies
IgG: immunoglobulin G
IPTG: isopropyl β-d-1-thiogalactopyranoside
SDS: sodium dodecyl sulfate
scFv: single-chain variable fragment
PAGE: polyacrylamide gel electrophoresis
VHH: variable heavy chain
